# Supplementary material for: Resolving the full spectrum of human genome variation using Linked-Reads
Source: Genome Res. 2019 Apr;29(4):635–45. doi: 10.1101/gr.234443.118 (PMC6442396; doi:10.1101/gr.234443.118)
Supplement: Supplemental Material [file supp_gr.234443.118_Supplemental_longranger-2.2.zip › longranger-2.2.tar.bz2!/lr2.2-public/lib/stan/cmdstan/stan/doc/api/html/structstan_1_1math_1_1index__type.html]

Stan: stan::math::index\_type< T > Struct Template Reference


|  |  |
| --- | --- |
|  | Stan  2.5.0  probability, sampling & optimization |


- Main Page
- Related Pages
- Namespaces
- Classes
- Files

- Class List
- Class Index
- Class Hierarchy
- Class Members

All Classes Namespaces Files Functions Variables Typedefs Enumerations Enumerator Friends Macros Pages

- stan
- math
- index\_type

stan::math::index\_type< T > Struct Template Reference

Primary template class for the metaprogram to compute the index type of a container.
More...

`#include <index_type.hpp>`

## Detailed Description

### template<typename T> struct stan::math::index\_type< T >

Primary template class for the metaprogram to compute the index type of a container.

Only the specializations have behavior that can be used, and all implement a typedef `type` for the type of the index given container `T`.

tparam T type of container.

Definition at line 21 of file index\_type.hpp.

---

The documentation for this struct was generated from the following file:

- src/stan/math/meta/index\_type.hpp


---

[ Stan Home Page ]

*© 2011–2014,
Stan Development Team.*
